# Moderate Heat-Assisted Gene Electrotransfer as a Potential Delivery Approach for Protein Replacement Therapy through the Skin

**DOI:** 10.3390/pharmaceutics13111908

**Published:** 2021-11-11

**Authors:** Chelsea Edelblute, Cathryn Mangiamele, Richard Heller

**Affiliations:** 1 Frank Reidy Research Center for Bioelectrics, Old Dominion University, 4211 Monarch Way, Suite 300, Norfolk, VA 23508, USA; cedelblu@odu.edu (C.E.); solsticepyrs@yahoo.com (C.M.); 2 Department of Biomedical Sciences, Graduate School, Old Dominion University, Norfolk, VA 23508, USA; 3 Department of Medical Engineering, Colleges of Medicine and Engineering, University of South Florida, Tampa, FL 33612, USA

**Keywords:** electrotransfer, gene delivery, electroporation, gene therapy, multi-electrode array, Factor IX

## Abstract

Gene-based approaches for protein replacement therapies have the potential to reduce the number of administrations. Our previous work demonstrated that expression could be enhanced and/or the applied voltage reduced by preheating the tissue prior to pulse administration. In the current study, we utilized our 16-pin multi-electrode array (MEA) and incorporated nine optical fibers, connected to an infrared laser, between each set of four electrodes to heat the tissue to 43 °C. For proof of principle, a guinea pig model was used to test delivery of reporter genes. We observed that when the skin was preheated, it was possible to achieve the same expression levels as gene electrotransfer without preheating, but with a 23% reduction of applied voltage or a 50% reduction of pulse number. With respect to expression distribution, preheating allowed for delivery to the deep dermis and muscle. This suggested that this cutaneous delivery approach has the potential to achieve expression in the systemic circulation, thus this protocol was repeated using a plasmid encoding Human Factor IX. Elevated Factor IX serum protein levels were detected by ELISA up to 100 days post gene delivery. Further work will involve optimizing protein levels and scalability in an effort to reduce application frequency.

## 1. Introduction

Gene electrotransfer (GET) holds great promise for the delivery of therapeutic agents. A number of clinical trials have shown positive results for gene-based therapies or vaccines [1,2,3,4,5,6,7,8]. Expression of the delivered gene should be controlled in order to achieve an effective therapeutic outcome while minimizing toxicity and other adverse effects. Electrotransfer has been demonstrated to be an effective approach for delivering plasmid DNA to a variety of tissues in vivo [9]. Due to the accessibility and ease of monitoring, skin is an excellent target for gene transfer protocols. The majority of gene-based approaches targeting the skin have focused on localized skin disorders or skin cancer [10]. There have also been multiple gene transfer applications that have demonstrated utility as potential treatments not only for cutaneous diseases but also for wound healing and ischemia [11,12,13,14,15,16,17,18,19]. The presence of antigen presenting cells has made the skin an excellent target for the delivery of DNA vaccines [20,21,22,23,24,25,26,27,28]. In addition, due to the presence of capillaries present in the dermal layer [29], skin can be an excellent target for gene therapies that seek to enhance protein levels within the blood circulation or potentially target distant organs [30,31,32]. Though effective, in order to penetrate the stratum corneum, cutaneous GET requires electric pulse conditions, including applied voltage or pulse number, that can result in discomfort or tissue damage. A minimally invasive method that does not require the use of high electric fields or can reduce the number of pulses would facilitate delivery with minimal local discomfort and could potentially reduce or eliminate visible scaring.

To enhance the translatability of GET, it is imperative to modify the approach in a manner that would result in reducing potential tissue damage and reducing or eliminating sensation associated with the pulse application while maintaining transgene expression. This could be accomplished by lowering the applied voltage or the number of pulses applied. With respect to delivery that can result in elevated levels of the expressed protein in the circulation, it is important to be able to achieve delivery in a minimally invasive manner to the papillary or reticular dermis or possibly to the muscle layer. One concept to achieve this is to reduce the distance between electrodes. We have previously shown the efficacy of a non-invasive multi-electrode array (MEA) for gene electrotransfer to the skin that utilizes a short gap between electrodes [20,33,34,35,36]. This concept has been applied successfully for delivery of DNA vaccines, treating ischemic tissue, or wound healing [17,37,38,39,40]. Although this approach improved the outcome with respect to reduced tissue damage, expression was still confined to the epidermis [34]. An additional advance developed has been to utilize this type of device to deliver to both muscle and skin [41], but this concept requires the use of invasive needle electrodes. Therefore, alternative strategies were explored to further improve the effect on tissue as well as to achieve deeper penetration while still utilizing noninvasive electrode arrays. GET is generally considered a nonthermal process wherein a high voltage pulse is applied, creating transient passage through the cell membrane for extracellular deliverables. However, the application of moderate heat can enhance cell membrane fluidity [42]. We therefore explored the combination of gene electrotransfer and moderate heating and demonstrated that this could be used to enhance gene delivery to cells and tissues [43,44,45].

1. Although increased temperature can enhance delivery when used together with GET, it is also important to moderate the applied heat so as not to cause tissue damage. It is well established that thermal injury is determined by temperature and duration [46] such that as the temperature is increased, the less time it takes to cause a burn or tissue damage. We previously established that the optimal intradermal temperature for enhancing GET was 43 °C [43,47]. It has also been accepted that it takes several minutes to cause a burn at 43 °C. A protocol was developed that demonstrated a 30 s preheating duration was sufficient for sustained temperature during the proceeding pulsing protocol. This entire process took less than a minute. Furthermore, the synergy between moderate heating and electroporation allows for the reduction of both the necessary applied voltage and pulse number [48]. These reductions have the potential to create an application that is less painful for the recipient and easier to apply for the clinician. Minimizing discomfort is an important consideration for translation of cutaneous deliveries where multiple applications may be necessary, such as protein replacement therapy.

Factor IX is a critical clotting protein present in human plasma. Patients suffering from Hemophilia B are variably deficient in the content or activity of this protein, and must receive repeated scheduled injections of intravenous Factor IX to survive [49,50]. Gene therapy could be an effective therapeutic approach as the defect is known and the patient could be treated by replacing the deficient protein [51,52,53]. The major target for gene therapy for hemophilia, whether using viral vectors or plasmid DNA have used the liver or muscle as the primary target [51,52,53,54,55]. Liver has an advantage being a natural site for production of FIX. Muscle is also advantageous as delivery can result in production of high quantities of the expressed protein for a long period. Gene therapy studies with adeno-associated virus (AAV) has progressed the furthest of the FIX replacement approaches that have been tested [51,54,55]. Skin is an attractive alternative for protein replacement gene therapy as the delivery could be performed in a minimally invasive manner.

We previously demonstrated the minimally-invasive delivery of Factor IX using the MEA, where the bulk expression was confined to the skin with a small portion reaching systemic circulation [56]. We thus evaluated if using moderate heating as an adjuvant to non-invasive GET may yield higher or more sustained Factor IX protein expression.

**Figure 1:**
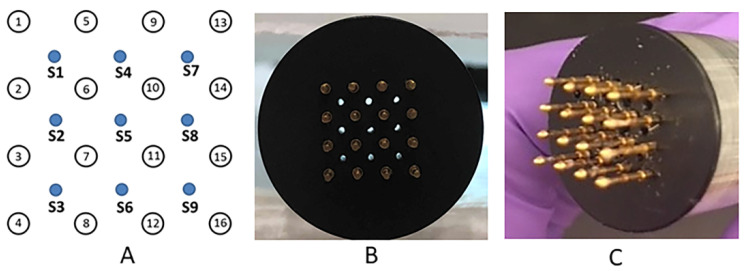
Moderate heat-assisted GET device. (**A**) The 16 pin MEA with evenly interspersed nine optical fibers encased within a Teflon cap. Each of the 16 pins is represented by circles with numbers. Holes between each cluster of 4 pins is where the fiber optic outputs are located (represented by blue circles labeled S1–S9). (**B**) Moderate heating is accommodated by an infrared laser split into nine equally spaced fibers (visible as holes). (**C**) Array from side angle showing clear view of the spring-loaded pins. GET pulses are applied from these spring-loaded pins that are placed in contact with skin target.

The current work demonstrates the utility of moderate heat-assisted GET to the skin using a novel application device based on the design of the MEA. Previous work demonstrating moderate heat assisted GET utilized a 4-pin electrode array designed as a 5 × 5 mm square with a single laser fiber placed in the center of the square [45,47,48]. With this new modified device, the addition of an exogenous heating component allows for enhanced uniformity in thermal distribution across the target by way of nine optical fibers connected to an infrared laser (Figure 1). Experiments were conducted using reporter genes as a proof of concept to demonstrate both the kinetics and expression distribution in vivo following moderate heat-assisted GET. In subsequent experiments, this combination platform was tested for cutaneous delivery of a therapeutic protein, human Factor IX. The aim of this work was to determine if moderate heat-assisted GET resulted in the expressed protein reaching the blood circulation and for how long this expression was maintained.

## 2. Materials and Methods

### 2.1. Animals

Animals used for this study were 8–10-week-old female Hartley guinea pigs weighing approximately 300–350 g purchased from Charles River Laboratories. The animals were housed at the Old Dominion University animal facility and procedures approved by the Old Dominion Institutional Animal Care and Use Committee at an AAALAC-accredited facility. IACUC protocol number 17-022 (original approval 12/19/2017) at ODU was followed in accordance for all procedures. All animal subjects were quarantined for a minimum of 7 days following arrival and prior to the conduction of any procedures. A total of 46 animals were used in this study.

### 2.2. Plasmids

Endotoxin-free plasmid preparations at 2 mg/mL in 0.9% sterile injectable saline of gWizLuc, encoding firefly luciferase, and gWizLuc-myc-DDK, encoding the oncogene myc and a DDK protein tag were used for this study. The gWiz-Luc-Myc-DDK plasmid was constructed using the sequence encoding luciferase from gWiz-Luc (Genlantis, San Diego, CA, USA) followed by in frame Myc-DDK tag OriGene (Rockville, MD, USA) and cloned into pBluescript II SK(+) (Biomatik, Wilmington, DE, USA) [57]. Both plasmids were commercially prepared for this study (Aldevron, Fargo, ND, USA). In addition, for testing a therapeutic gene, endotoxin-free plasmid preparation at 2 mg/mL in 0.9% sterile injectable saline of human Factor IX expression vector, pNGVL3-CMV-hFIXm1 (generous gift by Dr. Kurachi) was commercially prepared (Aldevron, Fargo, ND, USA). The three plasmids used in this study all have a CMV promoter.

### 2.3. Infrared Laser Heat Application

Animals were anesthetized with medical grade oxygen containing 2.5–3.0% isoflurane (Forane). Prior to treatment both flanks were shaved and washed with mild soap and water to remove loose hair and cleanse the skin of any abundance of oil. Moderate heating via a Class IV Laser power supply (LaserMate Group, Inc, walnut, CA, USA) via a laser optic fiber (Model: M79L005 Thor Labs Inc., Newton, NJ, USA) was applied immediately following a 50 μL 2 mg/mL intradermal injection of plasmid DNA to the pre-washed and shaven flank skin for a total duration of 30 s, after which the laser was switched off. All safety precautions were adhered to while the laser was in operation, including eye protection and barriers.

### 2.4. Measuring Intradermal Skin Temperature

Prior to utilizing the addition of moderate heat to augment expression obtained with GET, the time needed to raise the temperature of the dermis using an exogenous heat source in this novel configuration was determined. This was accomplished by inserting an 18G needle intradermally into either the left or right flank then carefully replacing with a K-type thermocouple temperature probe (Omega, Stamford, CT, USA). A baseline temperature of 35 °C was determined. With the thermocouple in place, the MEA with incorporated heat source was placed in contact with the surface of the skin above the temperature probe at a height of 1 cm, as pre-determined by the height of the electrodes. Temperature measurements were then obtained during the application of moderate heat. An intradermal temperature of 43 °C was achieved after operating the infrared laser across all nine fibers for 30 s at a power of 8 Watts. This temperature was maintained for approximately 20 s, thus allowing for the pulse protocol to be completed while the tissue remained moderately heated. These heating parameters were in-line with previously reported results from our group utilizing the same infrared laser though in an updated applicator design described below [47].

### 2.5. Electrode Design

**Figure 2:**
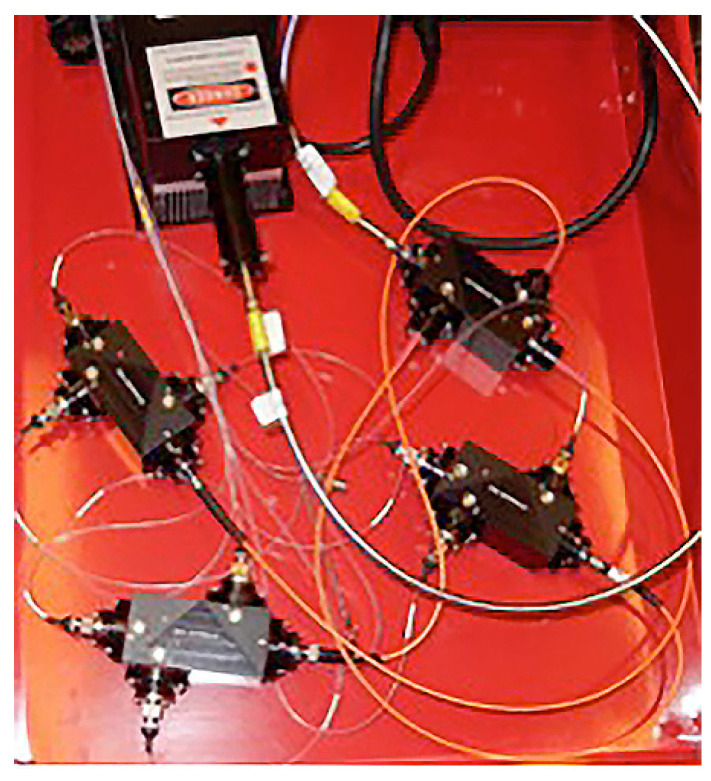
Infrared laser heating system. A main optical fiber for moderate heating connects to a primary three-way splitter box. Three secondary splitter boxes connect via three separate optical fibers to the primary three-way splitter box (orange fibers). Each of the three secondary splitter boxes directs the laser beam through three optical fibers connected to the main optical fiber. Shutter control is performed manually by nine individual shutters.

A novel applicator was constructed that enabled the administration of moderate heating and electric pulses. An infrared radiation emitter was integrated into the applicator. The emitter consists of an optical fiber whose beam is split equally among nine individual optical fibers utilizing four splitter boxes (Figure 2). The main optical fiber is directly connected to a three-way splitter box, which had three fibers each connecting to another three-way splitter box. Those nine fibers were then incorporated into the MEA (Figure 1A–C). These fibers are positioned equidistant and centrally between 16, 0.5 mm gold-plated round tipped pins each spaced 2 mm apart, creating a combined 6 by 6 mm moderate heating electrode array. As previously shown, the small size and dielectric material of the optical fiber can be integrated into a GET delivery system. The optical fiber was connected to an infrared semiconductor laser, providing up to 8 W of irradiative power at a wavelength of approximately 900 nm. The design of the electrode was modeled after the MEA, delivering 150 ms pulses applied in pairs perpendicularly where 4 of 16 electrodes are active at one given time [33,36]. Following this pulsing pattern, a total of 18 pulses are thereby delivered per round.

### 2.6. Reporter Gene Delivery

Animals were anesthetized with medical grade oxygen containing 2.5–3.0% isoflurane (Forane). Animals were pre-shaven and washed with mild soap and water to remove any loose hair or an overabundance of oil. A 100 μg intradermal injection of plasmid DNA was delivered to the flank. Injection sites were marked to ensure accuracy of data collection. The electrode array was immediately positioned over the injection area with or without exogenous moderate heating. For these studies both 2 and 4 rounds of 18, 150 ms pulses were delivered, yielding a total of 36 and 72 pulses, respectively. In addition, applied voltages of 35 V and 45 V were assessed. Gene expression levels were measured for all conditions by in vivo bioluminescent imaging or fluorescent imaging.

### 2.7. In Vivo Bioluminescent Imaging and Kinetic Expression Analysis

On days 2, 5, 7, 9, and 14, animals were anesthetized with medical grade oxygen containing 2.5–3.0% isoflurane (Forane) followed by a single subcutaneous injection of D-luciferin (Gold Biotechnology, St. Louis, MO, USA) at 150 mg/kg administered at the neck scruff. The animals were confined in an anesthesia chamber for eight minutes then transferred to the IVIS^®^ Spectrum (Perkin Elmer, Akron, OH, USA) imaging chamber under constant anesthesia. A circular region of interest (ROI) measuring 1.5 cm was selected on the image to encompass the entirety of the injection site and compared to untreated control ROIs. After background correction, bioluminescence results were represented as average total flux in photons/sec (p/s).

### 2.8. Immunofluorescence Staining and Distribution Expression Analysis

The localization of gene delivery within the skin was determined by immunofluorescence staining for the DDK tag protein. Skin samples were collected 48 h post gene transfer, fixed in 4% paraformaldehyde, paraffin embedded, and sectioned to six-micron thickness (IDEXX Laboratories, Inc., Westbrook, Maine, USA). Hematoxylin and eosin (H&E) staining on these samples were also performed on serial sections by IDEXX Laboratories. Unstained sections were deparaffinized using CitriSolv, and rehydrated in gradient alcohol (100, 95, 75, 50, and 25%). Antigen retrieval was performed in citric acid (pH 6). Cell permeabilization was performed for 20 min in 0.25% Triton X-100 in phosphate buffered saline (PBS). Blocking was performed with 4% bovine serum albumin in phosphate buffered saline with 0.01% Tween 20 (PBST) for 1 h at room temperature. Sections were then stained for immunoreactivity with DDK-tag protein with a mouse monoclonal anti-DDK antibody (TA50011–1, OriGene, Rockville, MD, USA) diluted 1:200 in blocking buffer overnight at 4 °C. Samples were then labeled with an AlexaFluor488 conjugated goat anti-mouse IgG secondary antibody (ThermoFisher Scientific, Grand Island, NY, USA). All samples were washed with PBST 5 times for 3 min on a shaker between antibody applications. Negative control samples were treated with secondary antibody only, without primary antibody. All staining procedures were performed in the dark. Samples were counterstained with DAPI for cell nuclei identification and mounted with VECTASHIELD^®^ HardSet™ mounting medium (Vector Laboratories, Burlingame, CA, USA) and allowed to set at room temperature. The samples were then stored in −20 °C until imaging.

### 2.9. Factor IX Gene Delivery

Animals were anesthetized with medical grade oxygen containing 2.5–3.0% isoflurane (Forane). As previously described, animals were pre-shaven and washed with mild soap and water to remove any loose hair or an overabundance of oil. Two 100 μg intradermal injections of plasmid DNA encoding human Factor IX were given on separate targets on the same flank. The electrode array was immediately positioned over the injection area with or without exogenous moderate heating. For these studies both 2 and 4 rounds of 18, 150 ms pulses were delivered, yielding a total of 36 and 72 pulses, respectively. In addition, applied voltages of 35 V and 45 V were assessed. Each site was injected, heated, and pulsed separately and each animal received only one pulsing condition with or without the application of moderate heat. An intramuscular GET group was included for comparison, an intramuscular injection to the hind limb was delivered followed by application of a 4-needle penetrating electrode delivering a total of 12, 20 ms pulses at 100 V/cm. The electrode measures 2.3 mm × 5 mm and pulses were given in trains of three via an ECM 830 (BTX, Holliston, MA, USA) adapted with a 4-position manual switch. These pulses were delivered at ambient temperature without the addition of moderate heating.

Factor IX gene delivery to multiple sites was carried out by prepping the animal subjects in the same way, with skin pre-shaven and washed whilst under a surgical plane of anesthesia. Multiple application sites were given in sets of 2, 3, or 4, 100 μg intradermal injections of plasmid DNA encoding human Factor IX. All sites were injected individually, immediately heated, then pulsed with 36 pulses at 45 V before proceeding to another site. Sites were evenly spaced along the same flank with 1.5 cm distance between sites. All sites received the same heating and pulsing conditions in this experiment where the scalability of Factor IX delivery was evaluated.

### 2.10. Factor IX Protein Expression Analysis

Blood from treated guinea pigs was collected via jugular vein puncture on days 2, 7, 14, 21, 35, 63, and 100. All collections were performed while animals were anesthetized with medical grade oxygen containing 2.5–3.0% isoflurane (Forane). Animals were monitored until recovery from anesthesia as evidenced by the ability to maintain sternal recumbency. Blood was allowed to clot for at least one hour at room temperature in serum separator tubes. Serum was carefully collected and used to measure clotting Factor IX content via a Human Factor IX ELISA kit (ab188393, Abcam, Boston, MA, USA).

### 2.11. Statistical Analysis

Statistical significance between groups for the reporter gene experiment was determined using a 2-way analysis of variance (ANOVA) with a Tukey-Kramer multiple comparisons posttest. Results are expressed as the mean of 6–8 replicates per group (± SEM). Significant results were determined with respect to animals receiving injection of plasmid DNA alone unless otherwise noted. A *p* value less than 0.05 was considered significant.

Statistical significance between the groups for the delivery of human Factor IX was determined by 2-way ANOVA with a Tukey-Kramer multiple comparisons test (GraphPad Prism Software, La Jolla, CA, USA). Results are expressed as the mean of five individuals per group (± SEM). A *p* value less than 0.05 was considered significant.

## 3. Results

### 3.1. Moderate Heat Applied by Infrared Laser Yields Fast and Uniform Heating

**Figure 3:**
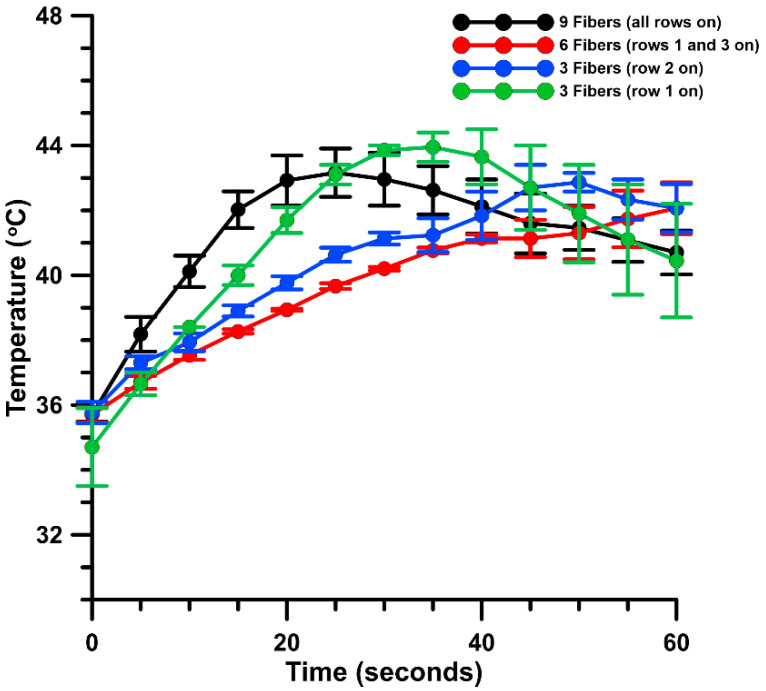
Intradermal skin temperature during moderate heating. In vivo intradermal temperature measured by thermocouple. Baseline temperature was recorded prior to each heating iteration: 3, 6, or 9 fibers. Moderate heating was applied via infrared laser positioned 1 cm away from the skin target. A target temperature of 43 °C was attempted for each heating condition, after which the exogenous heating source was turned off and intradermal temperature decay was measured. Temperature measurements were recorded manually at 5 s intervals for a total of 60 s. Average temperature is reported in degrees C per second ± SEM, *n* = 3 sites per group.

We initially tested the efficacy of tissue heating with this novel design. These data were collected via a thermocouple placed intradermally during the moderate heating protocol. A baseline intradermal temperature of 35 °C in the guinea pig skin was determined. Moderate heating to 43 °C utilizing all nine optical fibers took approximately 30 s (Figure 3). The temperature remained elevated above 41 °C for 20 s. There was no significant difference between all tested heating configurations: 3, 6, or 9 optical fibers.

### 3.2. Moderate Heat-Assisted GET Yields Sustained Expression Levels Compared to Unheated Counterpart

**Figure 4:**
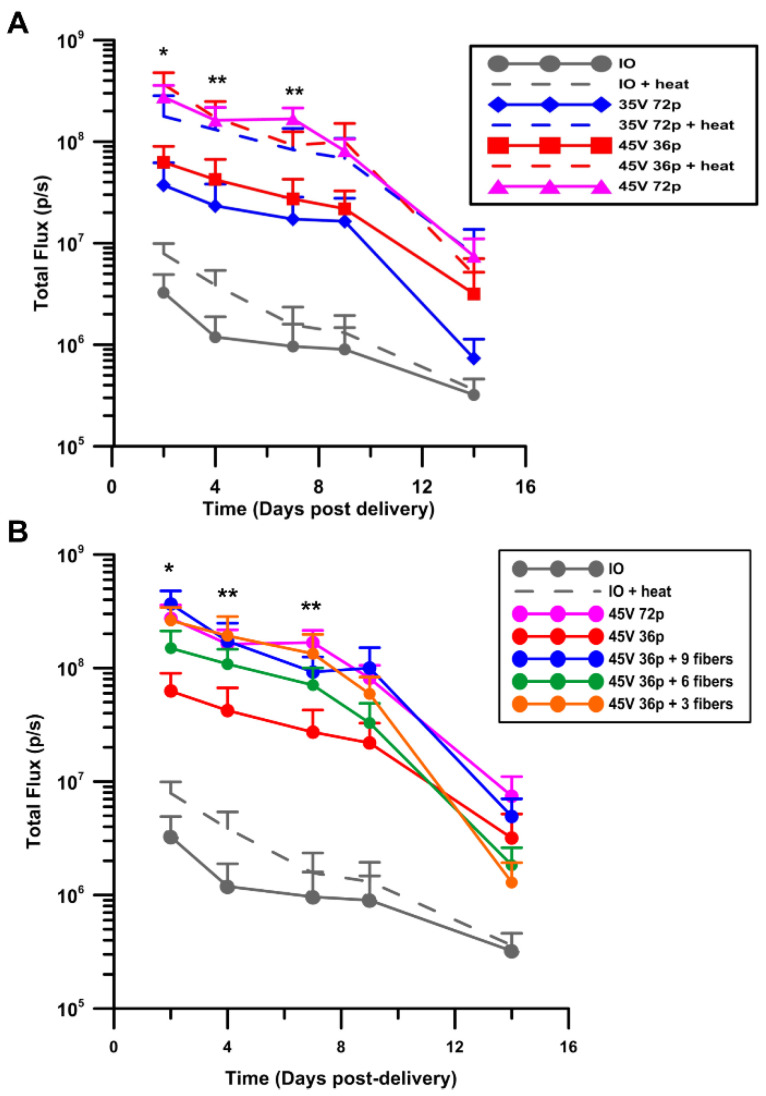
Kinetics of moderate heat-assisted GET to the skin. (**A**) Experimental groups included injection only (IO) plus heat (IO + heat), injection of pDNA followed by 72 pulses at 45 V (45 V 72p) or 35 V (35 V 72p), 72 pulses at 35 V with moderate heat (35 V 72p + heat), and 36 pulses at 45 V with (45 V 36p + heat) or without heating (45 V 36p). (**B**) Flexibility and distribution of heating was assessed for 3, 6, and 9 active optical fibers followed by GET at 45 V 36p. Luciferase expression levels reported as average total flux (photons/second) ± SEM, *n* = 6–8 individual sites per group. * *p* < 0.05, ** *p* < 0.01.

Moderate heat-assisted GET was demonstrated in a guinea pig model using a reporter gene as a preliminary tool to test gene delivery in the skin. The Luciferase expression pattern following the application of various pulsing parameters (Table 1) with and without the addition of moderate heat was recorded (Figure 4A). Peak expression measured in vivo was observed 48 h (the earliest recorded time point) after exposure in all tested conditions. The highest level of expression, 3.66 × 10^8^ photons/sec, was achieved with 45 V 36 pulses plus moderate heat at this time point. These results were significant with respect to an injection-only control (*p* < 0.05). Furthermore, this same condition yielded the highest expression levels on days 5 and 7 with a gradual decline over the 14-day observation period (*p* < 0.01). Results indicate that when preheating the skin to 43 °C prior to GET, the same expression levels could be achieved as GET without preheating but with a 23% reduction of applied voltage (45 V to 35 V) or a 50% reduction of pulse number (72 to 36 pulses).

We also determined the effect of reducing the number of active optical fibers for applying moderate heat to the target. As described, the infrared laser-heating component is distributed equally among nine optical fibers that are arranged equidistant in spans of three across three rows. They can be blocked individually or by row, therefore allowing for sharp control of the thermal distribution. We tested the efficacy of moderate heat row by row in combination with our highest performing GET condition- 45 V 36 pulses (Table 2). All of these tested heating conditions via 3, 6, or 9 optical fibers were significant with respect to an injection-only control at 48 h, 4 days, and 7 days post-delivery (*p* < 0.05, *p* < 0.01). However, there was no significant difference between applying moderate heat with 3, 6, or 9 optical fibers in our aforementioned highest expressing GET protocol (Figure 4B).

### 3.3. Gene Expression Following Moderate Heat-Assisted GET Extended to the Dermis and Underlying Muscle

**Figure 5:**
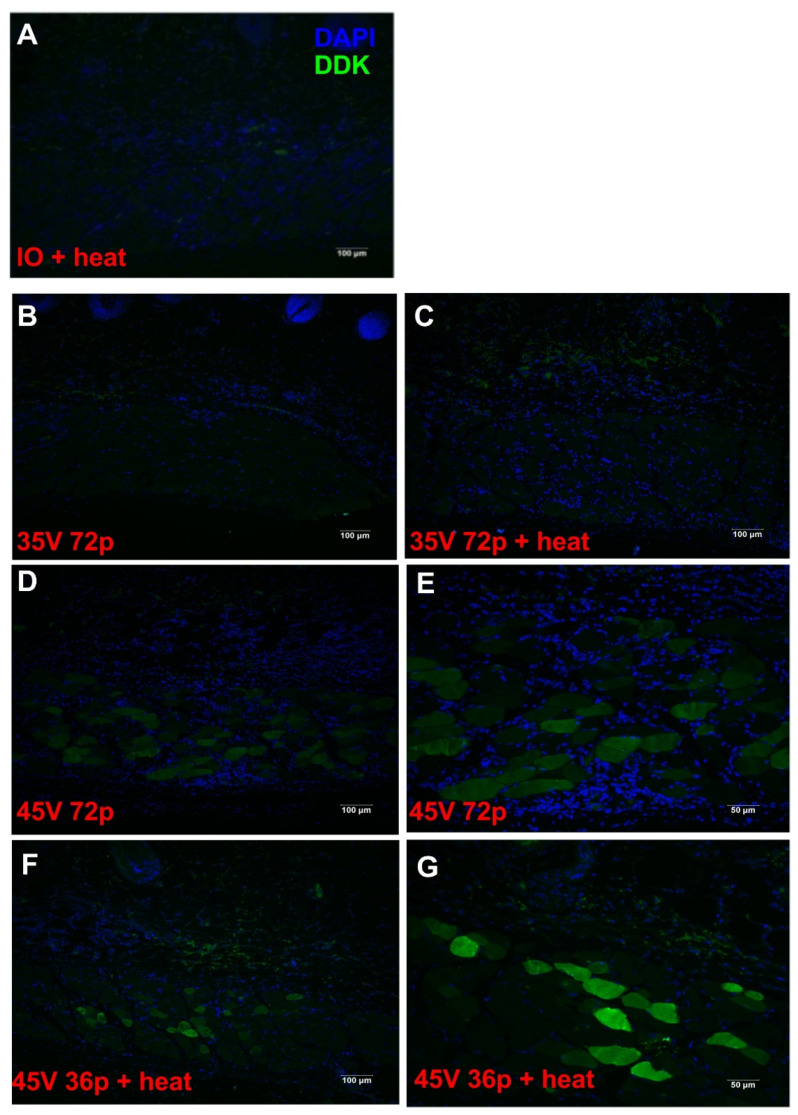
Expression distribution of moderate heat-assisted GET to the skin using gWizLuc-myc-DDK. (**A**) injection only plus heat (IO + heat); (**B**,**C**) injection of pDNA followed by 72 pulses at 35 V at ambient (35 V 72p) or after the application of moderate heating (35 V 72p + heat); (**D**,**E**) 72 pulses at 45 V (45 V 72p) applied at ambient; and (**F**,**G**) 36 pulses at 45 V after the application of moderate heat from an infrared laser (45 V 36p + heat). (**A**–**D**,**F**) 100×, scale bar = 100 μm. (**E**,**G**) 200×, scale bar = 50 μm.

To determine the distribution and localization of gene delivery following moderate heat-assisted gene electrotransfer, fluorescence staining was performed. The reporter gene, gWizLuc-myc-DDK, was delivered to guinea pig skin via intradermal injection with or without GET and moderate heat (Figure 5). No expression was observed following an intradermal injection with moderate heat alone (Figure 5A). Immunohistochemical evaluation demonstrated that expression was prevalent in the dermis and muscle layer of the tissue in those animals receiving moderate heat-assisted GET (Figure 5F,G). The addition of moderate heat resulted in a higher level of expression at the reduced 35 V compared to the same condition treated at room temperature (Figure 5B,C). When GET was combined with moderate heat using half the number of pulses, 36 (Figure 5F,G) versus 72 (Figure 5D,E), this expression pattern was more pronounced, suggesting enhanced delivery was achieved.

**Figure 6:**
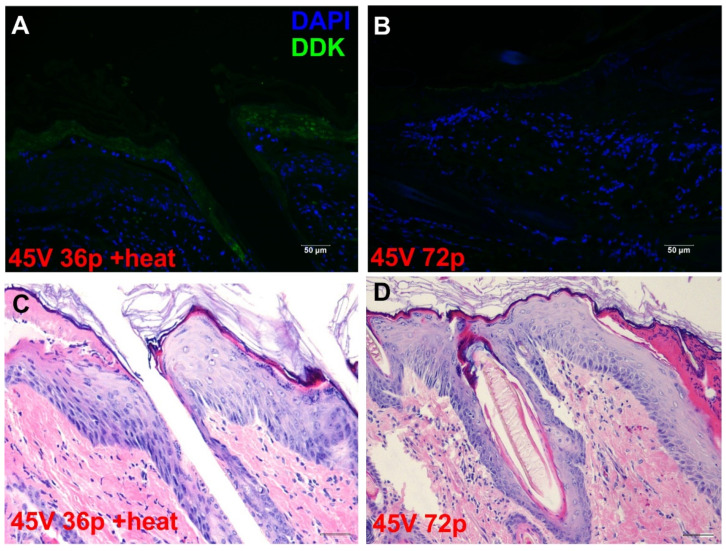
Expression distribution in epidermis after moderate heat-assisted GET to the skin using gWizLuc-myc-DDK. Images are representative of 3–4 individual sites. Epidermal DDK expression is shown in groups (**A**) injection of pDNA followed by 36 pulses at 45 V after the application of moderate heat (45 V 36p + heat) and (**B**) injection of pDNA followed by 72 pulses at 35 V at ambient temperature. 200×, scale bar = 50 μm. Corresponding H&E staining of epidermis for 45 V 36p + heat (**C**) and 45 V 72p without heat (**D**); 200× scale bar = 50 μm.

DDK expression levels evidenced by immunohistochemical evaluation at the epidermis were pronounced in those animals receiving moderate heat-assisted GET (45 V 36p + heat) (Figure 6A). Conversely, epidermal DDK expression was much lower in those animals receiving high-voltage high pulse GET at ambient temperature (Figure 6B).

### 3.4. Moderate Heating Mitigates a Reduction in Pulse Number and Skin Damage Caused by High Voltage GET

**Figure 7:**
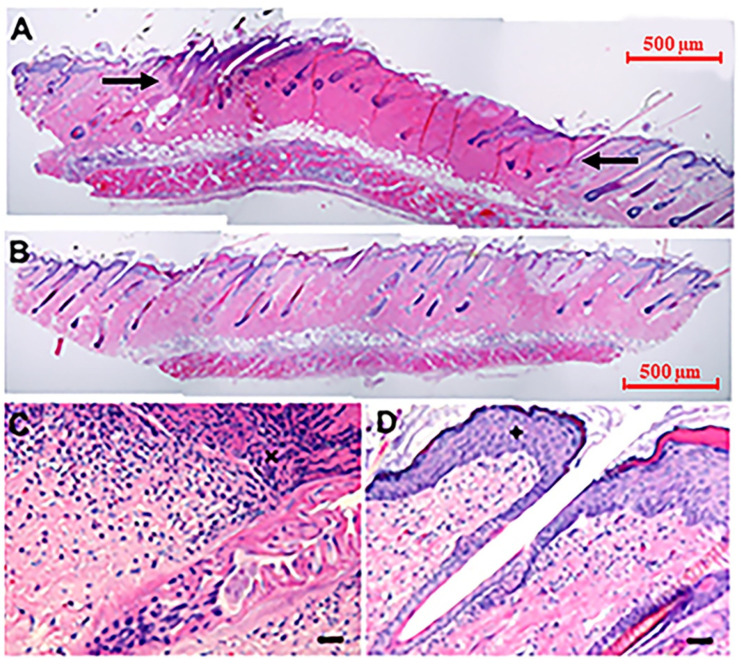
Histological analysis after cutaneous moderate heat-assisted GET 48 h after GET. Images are representative of 3–4 individual subjects. (**A**) injection of pDNA followed by 72 pulses at 45 V (45 V 72p no heat) between black arrows indicate areas of damage and (**B**) 36 pulses at 45 V after the application of moderate heat from an infrared laser (45 V 36p + heat). 40×. (**C**) Cell infiltrate visible at site of damage following 45 V 72 pulses, 200×, scale bar = 10 μm. (**D**) Intact tissue with no apparent damage after 45 V 36p + heat, 200×, scale bar = 10 μm.

Histological analysis was performed to assess tissue damage after the application of GET (Figure 7). H&E staining on fixed tissue sections from the expression analysis study harvested 48 h after gene delivery revealed that the application of a higher pulse number at a higher applied electric field resulted in a burn extending from the epidermis to the hypodermis with infiltrating cells suggesting injury (Figure 7A,C). When moderate heating was applied at the same electric field at 50% of the pulsing dose, histological damage was not observed (Figure 7B,D).

### 3.5. Factor IX Is Expressed Systemically Following Moderate Heat-Assisted GET to the Skin 

**Figure 8:**
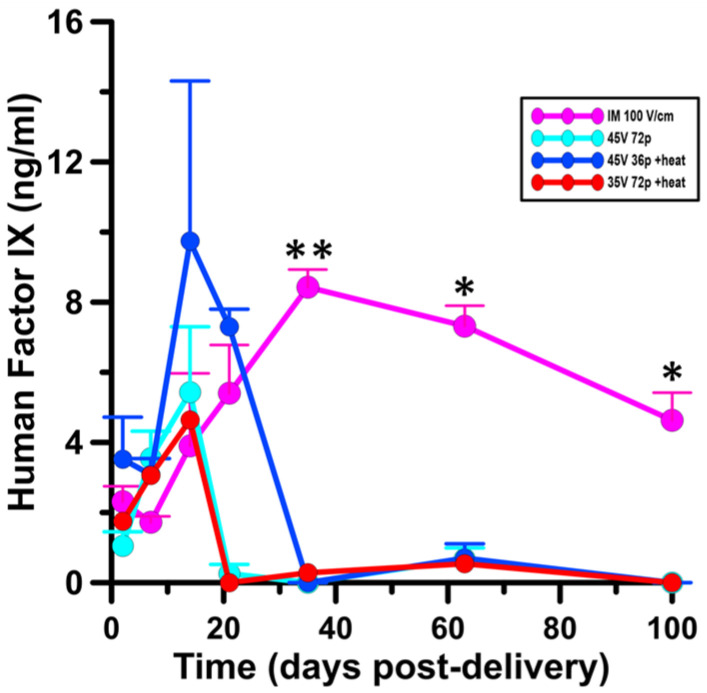
Factor IX expression following moderate heat-assisted and intramuscular GET. Human Factor IX in guinea pig serum. The data represent the average ± SEM, *n* = 5 individuals per group. * *p* < 0.05, ** *p* < 0.01.

We next tested for cutaneous delivery of a therapeutic protein, human Factor IX, in a guinea pig model. Serum was collected from animals following GET. Systemic Factor IX content was reported over a period of 100 days (Table 3; Figure 8). Moderate heat-assisted GET (45 V 36p + heat) yielded the highest protein levels nearing 10 ng/mL two weeks after delivery, at least 2- fold over any other treatment condition (Table A1). Expression waned for this condition and dropped completely by day 35. The other intradermal conditions, moderately heated or not, followed a similar protein expression pattern, though not at such a high level as the latter. We included an intramuscular GET (IM 100 V/cm) condition to serve as a positive control for Factor IX delivery. Conversely to the intradermal GET results, Factor IX protein levels following intramuscular delivery experienced an initial lag and peaked at 8.43 ng/mL ± 0.50 at 35 days (*p* < 0.01). This increased level of expression was sustained for another two months up to 100 days, ending the observation period.

### 3.6. Multiple Site Application Enhances Factor IX Protein Levels

**Figure 9:**
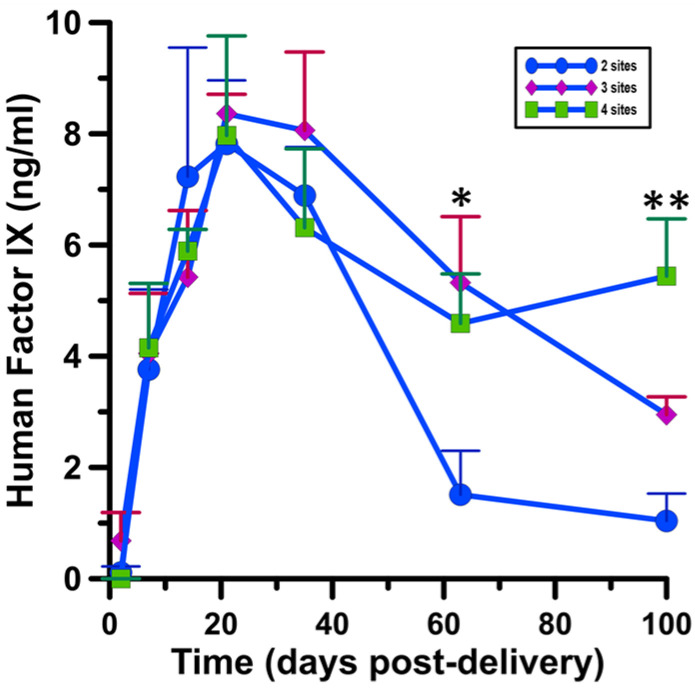
Scalability of Factor IX delivery via moderate heat-assisted GET. Human Factor IX in guinea pig serum. The data represent the average ± SEM, *n* = 5 individuals per group. * *p* < 0.05, ** *p* < 0.01.

The scalability of cutaneous delivery of human Factor IX via moderate heat-assisted GET was evaluated. Moderate heat-assisted GET was applied in sets of 2, 3, or 4 separate sites on the same flank in respective animal subjects (Table 4). Serum was collected from the animals following treatment. Systemic Factor IX protein levels were recorded over a period of 100 days (Figure 9). Utilizing our highest expressing delivery condition from the previous experiment, 45 V 36p + heat, we carried out multiple site applications. We observed the highest expression levels nearing 10 ng/mL 2–3 weeks after delivery across all three experimental groups (Table A2). Factor IX protein levels then began to wane. The most pronounced drop was in those animals receiving just two application sites, whereas those receiving three or four sites maintained elevated Factor IX protein levels for longer. Factor IX protein levels in those animals receiving three application sites was significant with respect to those receiving just two sites at day 63 (*p* < 0.05). Furthermore, in those animals receiving four moderate-heat assisted GET application sites, Factor IX protein levels in the serum were significantly elevated at days 63 and 100 compared to application of two sites (*p* < 0.05, *p* < 0.01).

## 4. Discussion

Heat has been used therapeutically since ancient times when Hippocrates recorded the advantages of fever in patients suffering from epilepsy [58]. Moreover, in 1927, the Nobel Prize in Medicine and Physiology was awarded to Julius Wagner-Jauregg for his work on the therapeutic value of fever therapy for neurosyphilis [59]. Today, topical therapeutic heat application falls under two categories: deep or superficial [60]. Deep heat, achieved with ultrasound or microwaves, reaches a penetration depth of 3–5 cm. This modality is preferred for treatment of deep-seated conditions such as bursitis or arthritis in the joints [60,61]. In contrast, superficial heat, such as delivered by electric heating pads, sodium acetate packs, or patches for minor injuries or sprains, is applied to achieve a penetration depth of less than 1 cm [62,63]. The results of both therapies are increased blood flow, elasticity of connective tissue, and, ultimately, pain relief. Although the use of therapeutic heat is quite commonplace, it is critical that both the temperature and duration of exposure be monitored to receive the beneficial effects without the damaging effects [63,64].

Previous studies have evaluated how cells and biological tissues are influenced by temperature changes, including the observation that there is an increase in cell membrane permeability in response to higher temperature [42,65,66]. This membrane dilation can facilitate the transfer of otherwise impermeable deliverables, for example, plasmid DNA or chemotherapeutic drugs to cells or tissues [45,47]. When the application of moderate heating and electric field is combined, these effects are more pronounced and delivery is more efficient. Moderate heating is also advantageous in the context of irreversible electroporation, where the applied heat sensitizes cancer cells to the applied electric field, thus making a more effective treatment [48,67,68].

There are a number of associated effects of thermal application to consider, including: sensation, skin discoloration, cellular processes, and injury [69,70]. In the current work, we present a platform for reversible electroporation, where cell viability is maintained [28]. We heat the skin to a moderate temperature of 43 °C for a total of 30 s and maintain an elevated temperature for an additional 20 s after the exogenous heat source is removed. At this temperature, 41–43 °C, the skin is temporarily warm and normal metabolism still proceeds. We observed no injury to the skin after moderate heat was applied. Conversely, high voltages and pulse numbers can lead to skin damage. We did observe this effect by way of reddened skin and confirmed it through H&E, in animals receiving 45 V and 72 pulses without moderate heat, suggesting that although this condition yielded a high level of expression, it was damaging and not ideal for translation in this application. The combination of moderate heating and GET allowed for a significant reduction in both pulse number and applied electric field all while achieving similar, or even higher overall expression levels in some cases. Moreover, tissue damage was not observed in reduced GET conditions with moderate heating. These are promising features of moderate heat-assisted GET, providing a shorter, less painful delivery platform compared to high voltage-high pulse GET alone. The result is a faster, minimally invasive route of electrotransfer. This is advantageous for applications such as protein replacement therapy where multiple deliveries and various schedules are necessary.

For this reason, we suggested the use of moderate heat-assisted GET for delivery of human clotting Factor IX. Factor IX is a plasma protein that is critical for thrombosis [71]. Hemophilia B is an X-chromosome linked genetic bleeding disorder in which patients are variably deficient in Factor IX, with ranges below the normal 50–100% plasma protein content [71]. It affects approximately one in 30,000 live births and 20% of all hemophiliacs. Current therapy for Hemophilia B requires frequent dosages administered via injection or central line. These formulations are either recombinant protein or plasma-derived product and have short half-lives. Plasmid DNA, in contrast to the aforementioned platforms, is self-stable, inexpensive to produce, and innocuous. In the last five years there have been viral gene therapy clinical trials using recombinant adeno-associated virus (AAV) to deliver Factor IX [71,72,73]. Though there has been some success in showing extended therapeutic benefit, use of this therapy in patients such as children, those with pre-existing immunity to AAV, and those with hepatitis have been excluded. To mediate this, transient immune suppression or a gain of function Factor IX variants have been suggested to alleviate the unintended side effects from using an AAV vector [74,75]. For these reasons non-viral alternatives should be considered. Moreover, a minimally invasive route of administration to the skin rather than to the liver, the natural site of Factor IX production, would be ideal in this clinical situation where the risk of severe bleeding is a real concern.

The high expression levels potentiate the use of moderate heat-assisted GET for protein replacement therapy. Sustained protein expression is critical for the treatment of conditions such as Hemophilia B, where less frequent visits and administrations are not only economical, but also lifesaving. We demonstrated that moderate heat-assisted GET yielded a significantly higher and longer duration of expression compared to needle-fitted syringe injection alone. In addition, both the pulse number and voltage were reduced by 50% and 23%, respectively, with the addition of moderate heat, thereby minimizing the perceived pain and superficial damage. Expression was localized in the epidermis, as well as the dermis and muscle layers, thus permitting the ability of therapeutic proteins to reach systemic circulation where they can be most effective.

We included an intramuscular GET delivery dose in our Factor IX experiment as a positive control, as this platform has been shown to yield long-term sustained transgene expression [76,77,78,79]. We observed just that in our study, with Factor IX protein levels peaking 35 days after IM GET delivery and waning gradually out to 100 days of continued observation. At day 35, expression among all intradermal delivery groups dropped. In comparing this pattern to our intradermal GET delivery, peak expression was observed 14 days following moderate heat-assisted GET at 45 V 72 pulses, where expression levels after IM GET were 2.5-fold lower at this same time point (*p* < 0.05).

Of note, these results show that we were able to achieve gene delivery to the muscle with an intradermal delivery approach using a non-penetrating electrode. Where we typically view an intradermal delivery platform as being confined to the epidermal and dermal cells, the addition of moderate heating allows for aqueous delivery via cutaneous GET to the hypodermis and muscle tissues. Our immunohistochemical results confirm this. Conversely, in IM GET, all of the plasmid DNA is injected into the hindlimb muscle, following application of pulses using a penetrating electrode. We therefore suggest that the utility of moderate heat-assisted GET in the skin for deep penetrating gene expression could be further studied and optimized.

Although these results are very promising, we do recognize that actual protein levels are still low. Normal physiological level of Factor IX is 100 ng/mL, so adjustments would need to be made to increase the amount of protein that actually reaches circulation, otherwise this could only be useful for patients suffering from mild Hemophilia B, where Factor IX levels are greater than 5% but less than 50% total plasma protein content. We addressed this concern by evaluating the scalability of moderate heat-assisted GET for Factor IX delivery. In this case, we selected our highest expressing GET condition (45 V 36p + heat) and tested the effects of additional sites on Factor IX protein levels in the serum. We observed peak protein levels in all experimental groups of 2, 3, and 4 injection sites within 21 days after initial delivery. Like our initial experiment, protein expression dropped sharply after peaking in those animals receiving just two application sites. Interestingly, protein levels remained significantly elevated in animals receiving 3 or 4 moderate heat-assisted GET application sites with prolonged Factor IX expression up to 100 days after delivery. This is encouraging and suggested the potential to scale up to enhance the amount of protein reaching systemic circulation. Not only was expression extended through 100 days, but the levels achieved during this time frame were equivalent to those achieved utilizing intramuscular delivery. This demonstrates that it is possible to achieve this outcome using a minimally invasive approach. Beyond simply adding more injection sites, modifications to increase the delivery surface area could be achieved by designing a larger applicator.

In another recently published work, we report that moderate heat-assisted GET can be used for the delivery of a DNA vaccine against Hepatitis B virus. After 18 weeks following a high-voltage high-pulse GET protocol with moderate heating (45 V 36p + heat), antibody titers were 230-fold higher than a moderately heated injection only control. Antibodies against Hepatitis B surface antigen remained elevated for 30 weeks following a prime pulse boost vaccination protocol with this platform [80]. These results combined with the Factor IX protein replacement therapy presented here, suggest there are a myriad of applications moderate heating could assist in enhancing the utility of current GET protocols.

Moreover, alternative therapeutic purposes where smaller amounts or weight of product are needed, such as growth hormone delivery, could also be an appropriate application for this technology [78,80,81]. An infrared laser was chosen as the heating source in this case due to its speed and precision at which the target temperature could be reached and specifically applied. However, utilizing different albeit slower exogenous heating sources such as LED, induction, or convection could be advantageous towards creating a more user-friendly platform in general.

## 5. Conclusions

Moderate heat-assisted GET provides efficient cutaneous gene delivery. Utilizing this approach can result in maintaining high expression for up to two weeks after a single delivery. The addition of moderate heat allows for a reduction in both the applied voltage and pulse number, all while achieving similar and in some cases higher gene expression. Utilizing moderate heat-assisted GET enabled increased gene transfer to dermal and muscle cells compared to GET alone. High pulse number GET was shown to result in irreversible damage to the skin, whereas moderate heat-assisted GET applied at the same voltage with half the amount of pulses did not cause skin damage. Systemic Factor IX expression was achieved following moderate heat-assisted GET. Intramuscular GET resulted in lagged, but sustained Factor IX expression up to 100 days. This result could be matched by intradermal delivery using moderate heat assisted GET by increasing the area delivered to. Further studies will focus on translating these findings into a two-armed therapy, combining both moderate heat-assisted GET and intramuscular GET, for long-term endogenous gene expression.

## Figures and Tables

**Table 1:** Kinetics of moderate heat-assisted GET—pulse parameters.

Group	pDNA	Heat	GET	Voltage	Pulse Number	N
IO	Yes	No	No	NA	NA	6
IO + Heat	Yes	Yes	No	NA	NA	6
35 V 72p	Yes	No	Yes	35	72	6
35 V 72p + Heat	Yes	Yes	Yes	35	72	6
45 V 36p	Yes	No	Yes	45	36	6
45 V 36p + Heat	Yes	Yes	Yes	45	36	6
45 V 72p	Yes	No	Yes	45	72	6

**Table 2:** Kinetics of moderate heat-assisted GET—fiber variation.

Group	pDNA	Heat	GET	Voltage	Pulse Number	N
IO	Yes	No	No	NA	NA	6
IO + Heat	Yes	Yes	No	NA	NA	6
45 V 72p	Yes	No	Yes	45	72	8
45 V 36p	Yes	No	Yes	45	36	6
45 V 36p + 9 Fibers	Yes	Yes	Yes	45	36	6
45 V 36p + 6 Fibers	Yes	Yes	Yes	45	36	6
45 V 36p + 3 Fibers	Yes	Yes	Yes	45	72	6

**Table 3:** Factor IX expression following GET with or without heat.

Group	phFIX	Heat	Injection Route	Voltage	Pulse Number	N
IM 100 V/cm	Yes	Yes	Muscle	23 & 50 *	12	5
45 V 72p	Yes	No	Dermis	45	72	5
45 V 36p + Heat	Yes	Yes	Dermis	45	36	5
35 V 72p + Heat	Yes	Yes	Dermis	35	72	5

* 23 Volts applied for 2.3 mm electrode gap and 50 Volts for 5 mm electrode gap.

**Table 4:** Scalability of Factor IX delivery.

Group	pDNA	Heat	Injection Route	Voltage	Pulse Number	N
2 Sites	Yes	Yes	Dermis	45	36	5
3 Sites	Yes	Yes	Dermis	45	36	5
4 Sites	Yes	Yes	Dermis	45	36	5

## Data Availability

All the data associated with this study are present in the paper. Materials are available upon request from the corresponding author.

## Appendix A

**Table A1:** Raw data used for Figure 8.

Animal #	Group	d2	d7	d14	d21	d35	d63	d100
19-0557	IM-100 V/cm	2.41	2.04	0	3.98	9.27	6.59	5391
19-0558	IM-100 V/cm	3.28	1.28	4.4	3.47	8.53	6.34	2.45
19-0559	IM-100 V/cm	1.15	1.66	1.71	9.48	8.91	7.46	4.47
19-0560	IM-100 V/cm	2.41	1.91	9.5	4.65	7.01	8.89	5.67
19-0561	45 V-72p	0	5.77	9.04	1.05	0	0	0
19-0562	45 V-72p	1.6	2.23	5.63	0	0	0.38	0
19-0563	45 V-72p	0.89	2.91	0.22	0	0	0.78	0
19-0564	45 V-72p	1.72	3.28	6.82	0	0	1.51	0
19-0565	45 V-36p + 9 fiber heat	6.85	4.29	0.85	7.79	0	0	0
19-0566	45 V-36p + 9 fiber heat	2.04	2.47	9.65	7.74	0	0	0
19-0567	45 V-36p + 9 fiber heat	1.53	3.28	22.34	7.87	0	1.62	0
19-0568	45 V-36p + 9 fiber heat	3.65	2.29	6.1	5.79	0	1.19	0
19-0569	35 V-72p + 9 fiber heat	3.03	3.21	9.6	0	0	1.14	0
19-0570	35 V-72p + 9 fiber heat	1.66	2.46	0.11	0	0	0.97	0
19-0571	35 V-72p + 9 fiber heat	1.28	3.34	3.16	0	1.12	0	0
19-0572	35 V-72p + 9 fiber heat	1.02	3.22	5.68	0	0	0.04	0

**Table A2:** Raw data used for Figure 9.

Animal #	Group	d2	d7	d14	d21	d35	d63	d100
19-1544	2 sites	0.42	0.85	3.99	4.79	6.21	0.38	0
19-1545	2 sites	0	1.77	4.21	7.46	8.8	0.82	1.64
19-1546	2 sites	0	5.74	6.8	9.06	4.83	1	0.45
19-1547	2 sites	0	6.67	13.92	9.98	7.71	3.83	2.06
19-1548	3 sites	0	1.41	3.27	7.46	8.46	4.84	3.4
19-1549	3 sites	2.17	3.2	4.77	8.41	9.5	7.98	3.37
19-1550	3 sites	0.54	5.99	4.78	8.39	10.29	6.12	2.97
19-1551	3 sites	0	5.61	8.87	9.17	3.97	2.33	2.04
19-1552	4 sites	0	1.7	5.33	2.94	6.34	4.09	8.04
19-1553	4 sites	0	3.11	5.34	7.87	2.29	2.36	3.42
19-1554	4 sites	0	4.7	5.9	10.27	8.19	5.47	4.21
19-1555	4 sites	0	7.1	6.99	10.79	8.43	6.45	6.09
